# Supplementary material for: Microarray Analysis Revealed Inflammatory Transcriptomic Changes after LSL60101 Treatment in 5XFAD Mice Model
Source: Genes (Basel). 2021 Aug 26;12(9):1315. doi: 10.3390/genes12091315 (PMC8468036; doi:10.3390/genes12091315)
Supplement: Supplementary file 1 [file genes-12-01315-s001.zip › genes-1305244-supplementary.pdf]

Table S1. Primers used in qPCR studies.

SYBR Green primers

Target	Forward primer (5′-3′)	Reverse primer (5′-3′)
Cxcr2	AGCCTTGAGTCACAGAGAGTT	CATCACCAAGGAGTTCCCCAC
Tlr5	GAATCCCGCTTGGGAGAACA	TTCCAAGCGTAGGTGCTCTG
Cd40lg	ACACGTTGTAAGCGAAGCCA	AACCGTCAGCTGTTTCCCAT
Ccl7	CCATCAGAAGTGGGTCGAGG	ACCATTCCTTAGGCGTGACC
Ifn-γ	CCTTCTTCAGCAACAGCAAGGCG	CTTGGCGCTGGACCTGTGGG
Ccr4	TGCTGGGTGGAGGAAATCAC	TCTCTACGCTTGTAACCAGGC
Sele	TAGCGCCTGGATGAAAGCAA	GAGCTCACTGGAGGCATTGT
Cxcl10	GGCTAGTCCTAATTGCCCTTGG	TTGTCTCAGGACCATGGCT
Ccl12	ACACTGGTTCCTGACTCCTCT	ACCTGAGGACTGATGGTGGT
Ccl8	GCCAGATAAGGCTCCAGTCA	TGCCTGGAGAAGATTAGGGGA
β-Actin	CAACGAGCGGTTCCGAT	GCCACAGGTTCCATACCCA

Supplementary Table S2

GENE ID	Wt Control	Wt LSL60101	5XFAD Ct	5XFAD LSL60101	p-value
Ccl1	1	1,032992102	1,15094422	1,884932204	n.s
Ccl11	1	1,270815528	1,14331882	1,191344106	n.s
Ccl12	**1**	**0,481851028**	**3,99291996**	**8,333132221**	$p<0.05$
Ccl17	1	1,190382143	1,277368	1,038318325	n.s
Ccl19	1	1,546786811	1,73005194	1,440855882	n.s
Ccl2	1	1,096755281	2,03153152	2,696859077	n.s
Ccl20	1	1,886762233	1,38726033	1,655926048	n.s
Ccl22	1	1,351568997	1,58656111	1,381677705	n.s
Ccl24	1	2,146417345	1,98937769	2,045137655	n.s
Ccl25	1	1,110255915	1,06345321	0,967076631	n.s
Ccl3	1	1,31004814	10,3301527	10,81069378	n.s
Ccl4	1	1,276080111	30,7110359	39,38441434	n.s
Ccl5	1	1,205309339	2,9608892	4,212315715	n.s
Ccl7	**1**	**1,429811808**	**0,84961387**	**1,426586791**	$p<0.05$
Ccl8	**1**	**0,975711901**	**0,92894392**	**1,739015967**	$p<0.05$
Cxcl1	1	1,839797674	1,94446271	1,903519007	n.s
Cxcl10	**1**	**1,138317491**	**2,19225813**	**3,71073393**	$p<0.05$
Cxcl11	1	4,659190833	3,70674761	2,205074164	n.s
Cxcl2	1	1,512042289	1,76824136	1,516368371	n.s
Cxcl3	1	1,259192236	1,19635538	1,410952841	n.s
Cxcl5	1	0,943196546	8,01424876	8,448143041	n.s
Cxcl9	1	0,92900287	0,90552645	0,969036067	n.s
Il10	1	1,266312426	1,04894235	1,278163656	n.s
Il17a	1	0,611364172	0,53441102	0,465842731	n.s
Il18	1	1,357317048	1,14683101	0,976779283	n.s
Il1a	1	0,907186054	4,52608795	3,768195125	n.s
Il1b	1	1,24868847	2,43569295	2,237394192	n.s
Il1rn	1	1,344395934	3,23980637	2,546518152	n.s
Il22	1	0,955646747	1,0655079	0,828987134	n.s
Il23a	1	1,071967888	1,20357685	1,298361223	n.s
Il5	1	1,720183242	1,12280886	1,112152514	n.s
Il6	1	1,038362254	1,29807631	1,27759769	n.s
Il7	1	0,967676455	0,82235136	0,740939002	n.s
Il9	1	1,356554612	1,36424338	1,106166724	n.s
Bcl6	1	1,020621229	0,88701838	0,855759965	n.s
C3	1	1,331562614	5,82015505	4,156739525	n.s
C3ar1	1	1,198281823	12,0069061	11,94679928	n.s
C4b	1	1,226794212	4,38557717	4,534341166	n.s
Cd14	1	0,957276703	6,26108156	6,39953496	n.s
Cd40	1	1,272562363	1,18706454	1,053353237	n.s
Cebpb	1	1,082372265	0,90663571	0,962606966	n.s
Crp	1	1,189001172	1,09635806	1,312109083	n.s

Supplementary Table S2

Fos	1	0,49460289	0,90756033	1,129720796	n.s
Itgb2	1	1,084690062	5,24672273	5,506719355	n.s
Kng1	1	1,2265623	1,29450477	1,336552177	n.s
Ly96	1	1,167852343	1,39330016	1,232392812	n.s
Nfkb1	1	0,988979556	1,35614935	1,345466348	n.s
Nos2	1	0,91836652	1,14061333	1,124735823	n.s
Nr3c1	1	1,239048545	1,03030928	1,038219493	n.s
Sele	**1**	**2,101176726**	**2,47348191**	**1,116294826**	$p<0.05$
Tlr2	1	1,103537473	6,55102848	8,881976866	n.s
Tlr5	**1**	**1,139424159**	**1,66807479**	**1,051876714**	$p<0.05$
Tlr7	1	1,128041774	3,25750587	4,219684358	n.s
Tlr9	1	1,341537379	1,47592438	1,510985171	n.s
Tollip	1	1,174904867	1,07298452	1,010877258	n.s
Cd40lg	**1**	**1,017940925**	**2,53177118**	**1,438604126**	$p<0.05$
Csf1	1	1,280000125	2,19985037	2,288264657	n.s
Lta	1	0,751911354	0,97912982	1,029406571	n.s
Il10rb	1	1,16277751	1,66685129	1,769717097	n.s
Il1r1	1	0,84701416	1,16916238	1,095703093	n.s
Il1rap	1	1,141661855	0,92852645	0,989515778	n.s
Il23r	1	3,838717085	4,15220271	3,067342221	n.s
Il6ra	1	1,207569456	1,56860982	1,469093194	n.s
Ccr1	1	1,461139927	2,73928657	2,091706017	n.s
Ccr2	1	1,014158219	1,60121897	1,372879324	n.s
Ccr3	1	1,094540484	0,93076087	1,135757463	n.s
Ccr4	**1**	**2,503131846**	**2,35409145**	**1,131088243**	$p<0.05$
Ccr7	1	1,092559803	1,23491921	1,132843686	n.s
Cxcr4	1	1,248939425	1,55674911	1,520328906	n.s
Cxcr1	1	1,256373653	0,34067734	0,329363975	n.s
Cxcr2	**1**	**1,317006598**	**3,59423804**	**1,341801673**	$p<0.05$
Tlr1	1	2,922283717	7,03857229	5,492933217	n.s
Tlr3	1	1,137350227	1,39984923	1,496429068	n.s
Tlr4	1	0,987095932	2,04836315	2,136494142	n.s
Tlr6	1	1,580604789	2,67363805	2,643446345	n.s
Myd88	1	1,123974745	1,41440615	1,379943449	n.s
Ripk2	1	1,135394145	1,07581686	1,196844218	n.s
Tirap	1	0,994621778	1,33022361	1,147671287	n.s
Fasl	1	0,979817436	0,90254562	0,993548033	n.s
Ifng	**1**	**2,399125108**	**0,88593312**	**1,695522811**	$p<0.05$
Ltb	1	1,226698523	1,385825	1,367220811	n.s
Tnfsf14	1	1,129229517	3,61732984	3,595060203	n.s
Tnf	1	1,66792704	4,29503733	3,526757083	n.s
Ptgs2	1	0,820924009	0,83421053	0,740363235	n.s
